# The emergence of metabolisms through Earth history and implications for biospheric evolution

**DOI:** 10.1098/rstb.2024.0097

**Published:** 2025-08-07

**Authors:** Edmund R. R. Moody, Tom A. Williams, Sandra Álvarez-Carretero, Gergely J. Szöllősi, Davide Pisani, Timothy M. Lenton, Philip C. J. Donoghue

**Affiliations:** ^1^ Bristol Palaeobiology Group, School of Earth Sciences, University of Bristol, Bristol, England, UK; ^2^ Bristol Organic Geochemistry Unit, School of Chemistry, University of Bristol, Bristol, England, UK; ^3^ School of Biological Sciences, University of Bristol, Bristol, England, UK; ^4^ GEE, University College London, London, UK; ^5^ Institute of Evolution, HUN-REN Centre for Ecological Research, Budapest, Hungary; ^6^ Model-Based Evolutionary Genomics Unit, Okinawa Institute of Science and Technology Graduate University, Kunigami District, Okinawa Prefecture, Japan; ^7^ Global Systems Institute, University of Exeter, Exeter, England, UK

**Keywords:** phylogenetics, comparative genomics, molecular clock, biosphere, metabolism

## Abstract

We investigate the evolution of microbial metabolisms from the last universal common ancestor to the extant biota through comparative phylogenomics, reconciling the evolution of the genes that underpin metabolic pathways with a time-calibrated tree of life. We find that the majority of metabolic pathways were established within the first 2 billion years of Earth history, with pathways accreting at different rates. Methanogenesis and acetogenesis are recovered to be among the earliest energy metabolisms, whereas photosynthetic pathways achieved completeness by 2 Ga, much later than most previous studies have envisaged. Horizontal exchange of metabolic genes is widespread, but it has occurred largely among closely related lineages and for some pathways there is a strong signal of vertical inheritance. We also find that the rate of horizontal gene transfer has been higher in Bacteria than in Archaea through evolutionary history. Finally, we evaluate how our reconstructed history of metabolism can help to constrain hypotheses of biospheric evolution, considering the entropic and Darwinized Gaia hypotheses as well as a simple neutral model for the assembly of biogeochemical cycles.

This article is part of the discussion meeting issue ‘Chance and purpose in the evolution of biospheres’.

## 1. Introduction

At its origin, our planet was lifeless, while today it hosts and forms part of a complex biosphere characterized by biogeochemical cycles that link Earth and life. The metabolic interplay among organisms drives the recycling of the core elements needed for life, collectively modulating Earth’s land, air, oceans and climate. This recycling is remarkably intense for essential elements including carbon, phosphorus and nitrogen, whose atoms are all cycled through primary production hundreds to thousands of times between entering and leaving the biosphere [1]. Life is also the main vector of nitrogen input to the biosphere. Understanding how life and the planet have co-evolved over time—how life is shaped by, and in turn shapes, the local and global environment, from the origin of life to the present day—is a core aim of Earth system science that promises to enrich our understanding of evolutionary history, the processes of ecology and evolution and how ecosystems function today to sustain our biosphere.

The primary challenge in elucidating this co-evolutionary history is calibrating the evolutionary history of life to Earth’s geological record. For most of Earth history, life has been entirely microbial, and today most biochemical and genetic diversity remains microbial. Plants and (to a lesser extent) animals influence contemporary biogeochemical cycling [2] but the major shifts in biospheric evolution were driven by the origin and evolution of microbial metabolisms, from the origin of life to the Great Oxidation Event in which oxygen produced by photosynthetic Cyanobacteria overwhelmed oxygen sinks, ultimately resulting in the oxidation of the atmosphere [3–6]. Microbes leave few interpretable fossils with which to link the origins of key metabolisms to geological time [7] and sedimentary geochemical records of metabolic activity can be difficult to link to the tree of life [8]. Consequently, the main source of information on the evolution of metabolism comes from comparative analyses of microbial genomes. The principal challenge to deciphering the evolutionary history of metabolisms is that the genes which underpin microbial metabolisms are prone to horizontal gene transfer (HGT), with useful genes potentially being transferred across large phylogenetic distances when microorganisms inhabit the same environmental niche [9]. This has led to suggestions that HGT scrambles the phylogenetic structure of microbial genomes over time, compromising attempts to trace the origin of key metabolisms [10,11]. Recent progress in methods development has focused on this challenge, with phylogenetic reconciliation models now available that aim to capture both the vertical and horizontal components of microbial genome evolution [12]. These methods have been used to reconstruct rooted species trees for Archaea and Bacteria and to draw inferences about ancestral metabolisms, including of the last universal common ancestor (LUCA) [13–16].

Here, we build on those analyses to reconstruct ancestral gene repertoires within prokaryote phylogeny, which allows us to trace the history of metabolic genes and the metabolisms they enable, from LUCA [17] to contemporary Archaea and Bacteria. By reconstructing gene repertoires at a series of time slices through evolutionary history, these analyses enable us to draw inferences about the sequence in which modern microbial metabolisms were assembled, and to deduce how the evolution of new metabolic genes has influenced the cycling of the core CHNOPS (Carbon, Hydrogen, Oxygen, Phosphorous, Sulfur) elements needed for life. Finally, we compare our reconstructed map to the predictions of alternative models of biospheric evolution [18,19], to ask whether empirical reconstructions of how life developed on Earth can be used to distinguish hypotheses about how biospheres emerge and are maintained over planetary timescales.

## 2. Material and methods

### (a) Timetree inference

We used MCMCtree (PAML v. 4.10.7; [20]) for divergence time estimation under the independent-rates log-normal (ILN [21,22]; and the geometric Brownian motion (GBM, or autocorrelated-rates model [23,24]) relaxed-clock models.

Our 700-taxa phylogeny (described previously as topology 2 in Moody *et al.* [16]) was inferred using a concatenation of 57 single-copy orthologous genes from 350 Archaea and 350 Bacteria, aligned with MAFFT (Multiple Alignment using Fast Fourier Transform) (L-INS-i) [25] and trimmed using BMGE (Block Mapping and Gathering with Entropy) (BLSM32) [26]. The final trimmed alignment contained 8152 amino acid sites. Individual and concatenated gene sequences and taxonomic information are available in our figshare repository [27]. This tree was time-calibrated by constraining the age of the following nodes based on established fossil evidence [16,28]. LUCA, crown groups: Chlamydia, Oxyphotobacteria (or Cyanobacteria); total groups: Eukarya (Heimdallarchaeota), Mitochondria (Alphaproteobacteria) and Oxyphotobacteria (or Cyanobacteria, including their non-photosynthetic relatives) and Chromatiaceae. In addition, total group Nostocales was constrained based on Davin *et al.* [29], and Thaumarcheota/Nitrososphaerota on the Great Oxidation Event (GOE). We also tested a new calibration for the total group of the archaeal clade including Euryarchaeota, TACK (Thaumarchaeota, Aigarchaeota, Crenarchaeota, and Korarchaeota) and Asgard Archaea, based on evidence for filamentous microfossils in combination with biogenic methane, for a hard minimum of 3331 Ma [30]. We performed four analyses, with and without this new calibration and under the two relaxed-clock models, to evaluate the robustness of the inferred timetree. Prior distributions and Markov chain Monte Carlo runs are described in detail in the accompanying Github repository at ‘anoxphoto-divtimes’ (https://github.com/sabifo4/anoxphoto-divtimes/).

### (b) Metabolic network over time

To estimate metabolic completeness across time, we used the reconciliation data from Moody *et al.* [16], with the divergence time estimates obtained in this paper (see above). Individual enzymes were determined as being ‘present’ where they had a probability of presence equal to or greater than 75%, and for the earliest time interval, we assumed the presence of the 399 gene families inferred to be present under the most stringent thresholds outlined in Moody *et al.* [16]. Using ipath v. 3 [31], we inferred the Kyoto Encyclopaedia of Genes and Genomes (KEGG; [32]) metabolic network over 500 Myr time slices until 1000 Ma. These data were then also used to generate pathway completeness (see below). To estimate rates of transfers and duplications over time, we used the time-calibrated branch lengths with the number of events from the same reconciliation to calculate the number of transfers per branch over time.

### (c) Metabolic pathway completeness

To estimate metabolic completeness across time, we used the same reconciliation data and divergence time estimates as above. We extracted gene contents for each tip of the tree and inferred ancestral gene contents for each node, then calculated the metabolic completeness of each of these gene repertoires using the ‘anvi-estimate-metabolism’ function (pathwise) in Anvi’o v. 8 [33]. We interpolated the completeness and age of each pathway between each mother and daughter node, using the mean age estimate. We then calculated the maximum completeness over 250 Myr time slices and plotted this with ggplot [34] in R [35].

## 3. Results

### (a) Growth of the metabolic network

**Figure 1.**
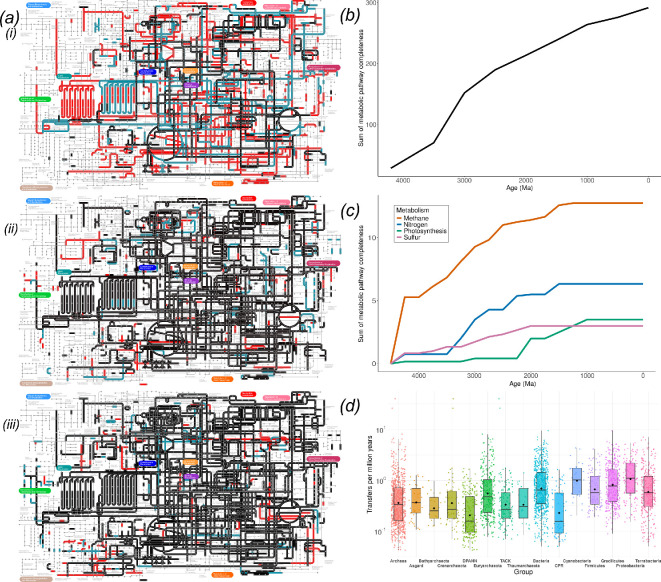
Growth of the total KEGG metabolic network over time. (***a***) The left panel plots the inferred assembly of the metabolic network through time with the oldest pathways in black, with younger pathways in teal and the youngest in red. (*i*) Starting with pathways inferred by 4 Ga (black), novel pathways by 3.5 Ga (teal) and by 3 Ga (red). (*ii*) Pathways established by 3 Ga (black), novel pathways by 2.5 Ga (teal) and 2 Ga (red). (*iii*) Pathways established by 2 Ga (black), novel pathways by 1.5 Ga (teal) and 1 Ga (red). (*b*) A line graph showing the increase in metabolic pathway completeness summed over time using the total network of gene families at the time slices in (*a*), with the maximum completeness for each KEGG module being 1 (for a maximum possible of 381 sampled pathways at the present-day). (*c*) Line graphs showing the increase in pathway completeness for four specific metabolism groups, methane (red, with a maximum completeness at present day of 13), nitrogen (blue, with a maximum completeness of seven), photosynthesis (green, with a maximum completeness of four) and sulfur (purple, with a maximum completeness of three). (*d*) A box plot representing the frequency of horizontal gene transfer over time, based on the sum of transfer events per branch per million years, for a range of prokaryotic groups. Individual data points are log-transformed (log_10_), and the *y*-axis reflects the transformed values. Boxplots show the median and interquartile range of log-transformed values. Means are indicated with black diamonds.

Moody *et al.* [16] reconstructed a species tree of prokaryotes and used phylogenetic reconciliation to calculate the probability that each of 7465 (out of a potential 9365) gene families found on modern archaeal and bacterial genomes trace back to the LUCA. Each family was assembled from the sequences of one KEGG orthologous group, such that the list of families present in LUCA could be used to reconstruct its metabolic capabilities. The analysis suggested that LUCA was a relatively complex anaerobic, rod-shaped, prokaryote-grade organism, capable of acetogenic growth using the Wood–Ljungdahl pathway and possessing an early immune system in the form of CRISPR-Cas proteins [16]. To estimate the growth of the metabolic network through time, we used the presence probabilities inferred for these 7465 gene families reconciled with the species tree used previously to infer the nature of LUCA and its descendant nodes [16]. Here, we build on this work by interrogating the inferred ancestral gene contents for each of the descendant nodes of LUCA on the same species tree. To determine the age of these ancestors, we used mean divergence time estimates for all nodes with a dated tree that evenly sampled 350 bacterial and 350 archaeal species [15]. We iteratively calculated the metabolic network at 250 Myr intervals starting at 4500 Ma (empty) to the present day (figure 1*a* shows the addition of novel families from 4 Ga to 1 Ga over three network panels). We inferred the evolutionary assembly of metabolic pathways by reference to KEGG [32].

The results show that much of the modern metabolic network characterized in KEGG—79% (303 out of 381) of pathways estimated to be present today (figure 1)—had already been established by 2.5 Ga, predating the GOE and the evolution of crown-eukaryotes [36,37]. The rate at which these pathways grew appears to be gradual and asymptotic (figure 1*b*,*c*). HGT played an important role in spreading new metabolisms among prokaryotes (table 1), with an average of 0.91 transfers per lineage per million years detected by the gene tree-species tree reconciliation analysis. However, HGT does not appear to have affected all metabolisms equally, and the inferred rate of HGT in deep time was significantly higher for Bacteria than for Archaea (figure 1*d*).

**Table 1.** The inferred earliest ages of key metabolic gene families for methanogenesis, nitrogen fixation, photosynthesis and dissimilatory sulfate reduction, reconciled with the dated species tree, HPD (Highest Posterior Density) in brackets, with the presence of probability at 0.75 and the respective domain to which the ancestral node belongs.

Name	Gene	Age (Ga)	Origin	Metabolism
Photosystem IP700 chlorophyll *a* apoprotein A2	psaB	4.47 (4.52−4.35)	LUCA	photosynthesis
Methyl coenzyme M reductase D subunit	mcrD	3.77 (3.98−3.56)	Archaea	methane
Photosystem P840 reaction centre iron–sulfur protein	pscB	3.12 (3.34−2.89)	Bacteria	photosynthesis
Nitrogenase iron protein	nifH	3.44 (3.68−3.18)	Archaea	nitrogen
Methyl-coenzyme M reductase beta subunit	mcrB	3.05 (3.39−2.67)	Archaea	methane
Nitrogenase molybdenum–iron protein beta chain	nifK	2.97 (3.21−2.75)	Bacteria	nitrogen
Nitrogenase molybdenum–iron protein alpha chain	nifD	2.89 (3.12−2.66)	Bacteria	nitrogen
Methyl-coenzyme M reductase gamma subunit	mcrG	2.64 (3.00−2.26)	Archaea	methane
Methyl-coenzyme M reductase subunit C	mcrC	2.64 (3.00−2.26)	Archaea	methane
Methyl-coenzyme M reductase alpha subunit	mcrA	2.53 (2.84−2.18)	Archaea	methane
Dissimilatory sulfite reductase alpha subunit	dsrA	2.17 (2.42−1.91)	Bacteria	sulfur
Dissimilatory sulfite reductase beta subunit	dsrB	2.17 (2.42−1.91)	Bacteria	sulfur
Photosystem II P680 reaction centre D1 protein	psbA	2.08 (2.33−1.91)	Bacteria	photosynthesis
Photosystem P840 reaction centre cytochrome c551	pscC	2.04 (2.27−1.82)	Bacteria	photosynthesis
Photosystem I P700 chlorophyll *a* apoprotein A1	psaA	1.59 (1.88−1.32)	Bacteria	photosynthesis
Photosystem II P680 reaction centre D2 protein	psbD	1.59 (1.87−1.32)	Bacteria	photosynthesis
Photosynthetic reaction centre M subunit	pufM	1.35 (1.55−1.16)	Bacteria	photosynthesis
Photosynthetic reaction centre L subunit	pufL	1.15 (1.35−0.97)	Bacteria	photosynthesis

### (b) Completeness through time

**Figure 2.**
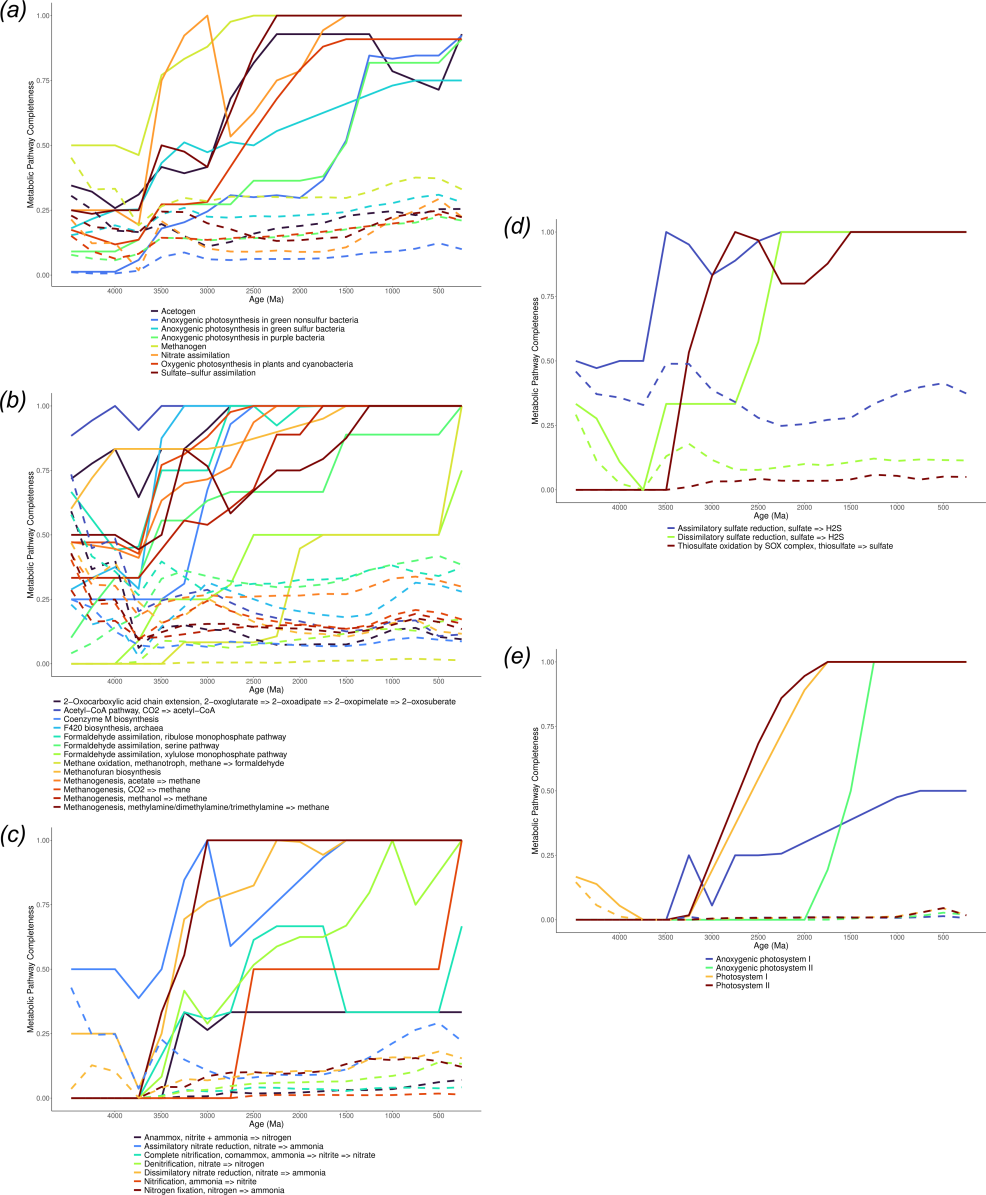
Completeness of selected metabolic pathways over time. Lines indicate the maximum metabolic completeness within a 250 Myr time bin, and dashed lines represent the mean completeness values across the same time bin. (*a*) Broad categories of energy metabolisms showing a general increase in their completeness over time. (*b*) Methane metabolism reflecting a range of different pathway trajectories, with an early origin of the acetyl-CoA pathway contrasted with the very recent development of methanotrophic pathways. (*c*) Nitrogen metabolism reflecting a spike in activity between 3.5 and 3 Ga. (*d*) Sulfur metabolism showing the early rise of assimilatory sulfate reduction before 3.5 Ga followed by thiosulfate and dissimilatory sulfate reduction before 2 Ga. (*e*) Photosynthetic metabolic pathways showing a rise in photosystem I and II from 3.5 Ga onwards, with anoxygenic photosystem II reaching completion between 2 Ga and 1.5 Ga.

In order to gain an understanding of the timing and tempo of evolutionary assembly of metabolic pathways, we used the maximum metabolic completeness of different KEGG pathways across time bins of 250 Myr (figure 2; for plots of all sampled metabolisms, see the electronic supplementary material on Figshare [38]. The link between pathway completeness and pathway activity is only indirect, and so it is challenging to determine precisely when a given metabolic pathway first emerged from these analyses. This is both because gene family functions can evolve over time, and because even an incomplete pathway can in principle be active so long as a subset of key enzymes are present. Given finite sampling of Archaea and Bacteria (350 representatives of each chosen for phylogenetic breadth in our analysis), the evolution of pathways that are widespread in modern taxa will be reconstructed in greater detail than those that are sparsely or patchily distributed. Our results show a gradual rise in the completeness of all major metabolic capacities, with the exception of rapid increases in the completeness of the nitrate assimilation (M00615) and methanogen (M00617) signature modules (sets of genes organized into functional units characterizing phenotypic features) [32] before 3.5 Ga (Eoarchean–Palaeoarchean; figure 2*a*), congruent with the earliest major diversifications of the bacterial and archaeal domains [36]. The most pronounced expansion of the metabolic network occurs in the Archean, between 3.5 and 3.0 Ga (figure 1). By 3.25 Ga, modern pathways for both nitrate assimilation (M00615) and methanogenesis (M00617) were almost fully established (figure 2*a*). However, it is not until the Palaeoproterozoic (2.5 Ga) that sulfate-sulfur assimilation (M00616) and oxygenic photosynthesis (M00611) reach 50% completeness. Two of the three anoxygenic photosynthesis signature modules (M00613, M00614) achieve greater than 75% completeness between 1.25 and 1.0 Ga (late Mesoproterozoic), post-dating the evolution of oxygenic photosynthesizing bacteria and eukaryotic photosynthesizers (Archaeplastida) [39]. Anoxygenic photosynthesis in purple bacteria (M00612) is later, peaking during the Neoproterozoic, although it should be noted that all the anoxygenic photosynthetic signature modules are inferred to be partially complete (25% completeness) by 3 Ga.

A breakdown of the specific pathways comprising methane metabolism (map00680; figure 2*b*) reveals that methanotrophic methane oxidation is inferred to be relatively recent, with the earliest emergence of any of the required enzymes dated between 3.25 and 3.0 Ga (Mesoarchean). Fifty per cent completeness is attained much later, between 1.75 and 1.5 Ga (late Palaeoproterozoic to earliest Mesoproterozoic). The acetyl-CoA pathway is the earliest methane-related pathway inferred to be active, showing almost 100% completeness in the first 250 Myr time bin. Other methane-related metabolisms such as formaldehyde assimilation, coenzyme F420, methanofuran biosynthesis and methanogenesis pathways broadly follow a similar pattern, with varying rates of increase in completeness from LUCA (Hadean) to 2.5 Ga (Archean-Proterozoic transition).

Abiotic sources of biologically available nitrogen could have been available relatively soon after the formation of the Earth [40,41] and may explain how early life [16] was able to incorporate nitrogen before the evolution of nitrogen fixation. However, a limited supply of biologically available nitrogen may have acted as a bottleneck in the early biosphere [42]. Assimilatory nitrate reduction is inferred to be at least partially present (50% completeness) in LUCA and is the first of the nitrogen metabolic pathways (map00910) to evolve. This was followed by the emergence of dissimilatory nitrate reduction and then nitrogen fixation between 3.25 and 2.5 Ga. Modern nitrification and denitrification pathways took longer to fully emerge, attaining 50% completeness before 2.25 Ga. Anammox and comammox (complete ammonia oxidation) are less clear, anammox peaks 3.25−3.0 Ga, but does not increase any more than this before the modern day, whereas comammox peaks 2.5−2.0 Ga, but appears to decline before another peak at 500 Ma (figure 2*c*).

Similarly, assimilatory sulfate reduction (M00176) also appears to be an ancient pathway (figure 2*d*), being 50% complete at the time of LUCA, and reaching a complete modern pathway at 3.5 Ga (Palaeoarchean). Thiosulfate oxidation (M00595) begins to emerge at 3.25 Ga, peaking at 2.25 Ga (Palaeoproterozoic). Our estimates of dissimilatory sulfate reduction (M00596) go from 50% at 2.5 Ga to a complete pathway by 2 Ga (mid-Palaeoproterozoic) (see below).

Photosynthesis can be achieved either through anoxygenic (anoxygenic photosystem I: M00598; anoxygenic photosystem II M00597) or oxygenic means (photosystem I: M00163; photosystem II: M00161) (figure 2e). Oxygenic photosynthesis sees a steep rise from 3.25 Ga to full completeness at 2 Ga for both photosystems. Elements of anoxygenic photosystem I appear to be present before the increase in oxygenic photosystems, while anoxygenic photosystem II rises in completeness during the Mesoproterozoic. However, anoxygenic photosystems are distributed patchily across modern Bacteria, and we note that a more detailed investigation of the history of these genes would require deeper sampling of the modern groups that encode them.

### (c) Origins of key metabolic enzymes

#### (i) Methanogenesis

**Figure 3.**
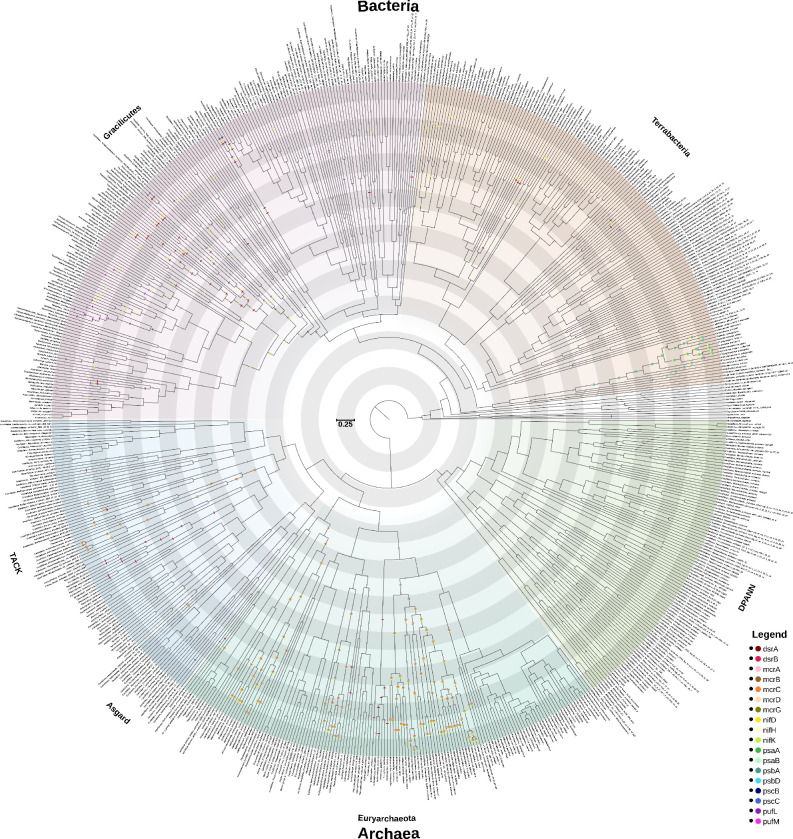
A time-calibrated tree of prokaryotes, showing the presence of enzymes in key metabolic pathways. Sensitivity analyses suggest that the inferred time tree is robust to a range of choices of clock model and calibrations (figure 4). This graph shows the inferred ancestral presence of key enzymes with the presence of probability ≥0.75 involved in dissimilatory sulfate reduction: dsrA (maroon), dsrB (red); methanogenesis: mcrA(pink), mcrB (brown), mcrC (orange) mcrD (apricot), mcrG (olive); nitrogen fixation: nifD (yellow), nifH (beige), nifK (lime); photosystem I: psaA (green), psaB (mint); photosystem II: psbA (teal), psbD (cyan); anoxygenic photosynthesis: pscB (navy), pscC (blue), pufL (purple), pufM (magenta). Terrabacteria (orange), Gracilicutes (pink), DPANN (Diapherotrites, Parvarchaeota, Aenigmarchaeota, Nanohaloarchaeota and Nanoarchaeota) (green), TACK (teal), Asgard (darker blue), Euryarchaeota (lighter blue). Shading and scale bar represent 0.25 Ga. High resolution version of image available in electronic supplementary material [44].

Methanogenesis is a key pathway in the modern global carbon cycle. Archaeal methanogenesis revolves around the use of the methyl–coenzyme M reductase complex (mcr) [43]. We inferred the presence of the mcrD subunit at 3.77 Ga (Eoarchean). McrB, appears 720 million years later (3.05 Ga), with the rest of the modern mcr complex evolving by 2.54 Ga, indicating that modern methanogenesis had evolved before the GOE (figure 3).

#### (ii) Sulfur

Microbial dissimilatory sulfate reduction is an important component of today’s sulfur cycle, facilitating pyrite formation, organic matter remineralization and contributing to marine carbon cycling. It plays an important role in maintaining redox balance in the ocean, and influences other biogeochemical cycles such as the carbon cycle [45,46]. Two key genes involved in dissimilatory sulfate reduction are dsrA and dsrB, they are also responsible for the reverse reaction of oxidizing sulfide to sulfite. However, our results indicate assimilatory sulfate reduction (M00176) predates dissimilatory sulfate reduction (M00596) (figure 2*d*) by almost 1 billion years. Our enzyme-focused analysis reveal that dsrA and dsrB originated at 2.17 Ga (HPD: 1.91−2.42 Ga), in the aftermath of the GOE (figure 3).

#### (iii) Nitrogen

Biological nitrogen fixation today, i.e. the production of ammonium ions (NH_4_^+^) from nitrogen gas (N_2_) is achieved through a family of enzymes called nitrogenases, which can be divided into subfamilies based on the metal cofactor (molybdenum-iron, vanadium-iron, or iron-iron) required to catalyse the reaction. Why only these three metals are used is uncertain [47]. Our individual enzyme analysis (figure 3) focuses on the molybdenum-iron nitrogenases (nifH, nifK and nifD). Although anfG (iron-iron nitrogenase) was included in the dataset, the only nodes where its probability of presence was above 0.75 were (modern-day) tips: *Methanosarcina acetivorans* (GCA_000007345), *Rhodopseudomonas palustris* (GCA_000014825), *Methanomassiliicoccus luminyensis* (GCA_000308215) and *Propionispora vibrioides* (GCA_900110485). Not surprisingly, the reconciled anfG trees were inferred to have had a high number of transfer events (>0.60) in all taxa with the gene. As no vanadium-based nitrogenases were included in the dataset [16], they were not included here. However, recent research has shown vanadium nitrogenases evolved much more recently than other types [48–50]. The origin of nifH significantly predates other molybdenum-based nitrogenase genes (nifK, nifD) by around 500 million years (table 1). Taken together, the results of our metabolic completeness (figure 2*c*) and individual enzyme (figure 3) analyses indicate that nitrogen fixation evolved in the mid-late Archean, around 3 billion years ago.

#### (iv) Photosynthesis

The key components of photosynthesis are the photosynthetic reaction centres. Although we infer the presence of psaB first, in LUCA, this is most likely an artefact from the homologous sequence found in a deeply branching bacterial taxa *Dictyoglomus turgidum*, given that after LUCA and LBCA we do not see psaB return until the common ancestor of Oxyphoto/Cyanobacteria, along with psbA (2.08 Ga), and both descending nodes then also containing the corresponding psaA and psbD (1.59 Ga) (figure 3). The anoxygenic photosystems also appeared to evolve early, with the pscB protein emerging within Grailicutes (around 3.12 Ga), with other components inferred to be present more recently: pscC (2.04 Ga), pufM (1.35 Ga) and pufL (1.15 Ga).

## 4. Discussion

### (a) Metabolic evolution

Previous work suggests that a small number of key elements of metabolic networks most likely existed before the diversification of life [51,52]. However, the majority of key metabolic pathways appear to have emerged since LUCA [8]. Our results suggest that although initial increase in the global metabolic network (figure 1*a*) happened relatively quickly (79% of pathways were already present before 3 Ga), the rate at which additional nodes are added to the network is relatively gradual (figure 1*b*,*c*).

Our analysis confirms that HGT has been common throughout the history of life (figure 1*d*), but that the spread of metabolic pathways may be more constrained than has been appreciated hitherto [10,11].

HGT occurs but, observationally, there appear to be limits on how these pathway elements can be transferred across the tree of life. We already know that the rate of HGT varies by functional category [53], but our results also show that some metabolic pathways show a degree of phylogenetic endemism through deep time. That is, despite ongoing HGT throughout life’s evolution, some sets of genes (beyond the core information processing machinery of the cell) tend to remain associated with particular lineages through deep time (figure 3). This suggests that the scrambling of gene–lineage associations during early evolution was not as thorough as it first appeared in genome-scale analyses using simpler phylogenetic methods that did not jointly consider species and gene trees [10,11]. Nevertheless, our results confirm that gene transfers have been very frequent (a mean estimate of 0.91 transfers per lineage per million years) and, since our analysis considers only long-distance transfers between relatively sparsely sampled lineages, we expect transfer among closer relatives to be more frequent still. We find that rates of HGT vary significantly across the prokaryotic tree (figure 1*d*; table 2), with HGT more frequent in Bacteria (1.09 transfers per lineage per million years) than in Archaea (0.72 transfers per lineage per million years). A potential explanation for this high-level pattern is a reduced exposure of Archaea to exogenous DNA as a result of the extreme or nutrient-poor environments in which many archaeal lineages persist [54], or the domain-specific challenges faced by archaeal viruses [55]. The highest rates of transfer and duplication were observed in Proteobacteria (a mean of 1.52 transfers and 0.26 duplications per million years) and Cyanobacteria (a mean of 1.34 transfers and 0.36 duplications per million years). Euryarchaeota similarly had a high rate of transfer (a mean of 0.97 per million years, but far fewer duplications: 0.19). The ecological opportunity to gain genes could also be an important factor, since host-associated clades have the lowest rates across Archaea (DPANN, mean: 0.32 transfers per million years) and Bacteria (CPR (candidate phyla radiation), mean: 0.38 transfers per million years).

**Table 2.** Mean number of transfers and duplications per million years for a selection of prokaryotic clades. (Mean results with an asterisk (*) are including two extreme crenarchaeal outliers, without these outliers, the mean for Archaea becomes 0.63, Crenarchaeota 0.43 and TACK: 0.42.)

Group	Transfers per million years (95% HPD)	Duplications per million years (95% HPD)
Archaea	0.72* (0.04–2.26)	0.13 (0.00–0.87)
Bacteria	1.09 (0.05–3.66)	0.18 (0.00–1.01
Asgard	0.47 (0.11–1.12)	0.18 (0.00–0.80)
Bathyarchaeota	0.34 (0.12–0.99)	0.04 (0.00–0.13)
Crenarchaeota	1.35* (0.07–1.64)	0.11 (0.00–0.56)
CPR	0.38 (0.06−1.21)	0.05 (0.00–0.31)
Cyanobacteria	1.34 (0.20–4.34)	0.36 (0.01–1.36)
DPANN	0.32 (0.04–1.07)	0.04 (0.00–0.27)
Euryarchaeota	0.98 (0.08–3.82)	0.19 (0.00–1.01)
Firmicutes	0.93 (0.19–2.78)	0.17 (0.01–0.76)
Gracilicutes	1.23 (0.12–3.99)	0.19 (0.00–1.07)
Proteobacteria	1.52 (0.15–4.06)	0.26 (0.00–1.16)
TACK	0.82* (0.07–1.37)	0.10 (0.00–0.54)
Terrabacteria	0.97 (0.05–3.21)	0.18 (0.00–0.98)
Thaumarchaeota	0.44 (0.08–1.41)	0.15 (0.00–0.56)

### (b) Fit to geochemical record

**Figure 4.**
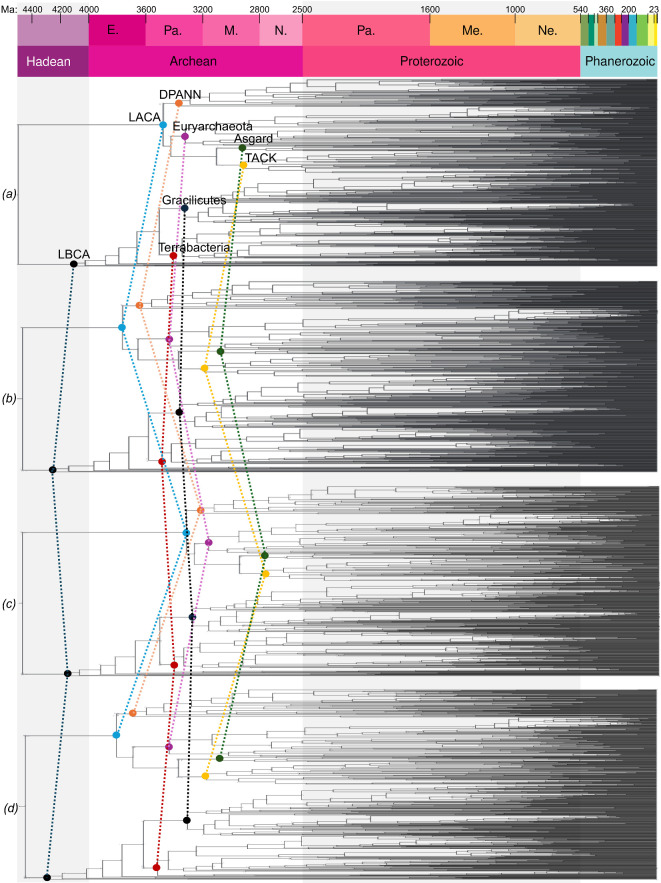
The effect of different calibration and model strategies on timetree inference. Divergence times of key nodes (highlighted) were only moderately affected by the choice of clock model or inclusion of a new calibration on the total group of Euryarchaeota, TACK and Asgard Archaea, based upon the earliest record of biogenic methane. With the deep archaeal calibration under a (*i*) GBM model; (*ii*) ILN model; or without the deep archaeal calibration under (*iii*) GBM and (*iv*) ILN models.

Where sedimentary geochemical evidence of metabolic activity exists, there is generally good agreement with our inferences (figure 3), which generally appear robust to a range of choices of clock models and calibrations (figure 4). There is coherent evidence for oxygenic photosynthesis by 2.945 Ga [56,57] and so our inference, that modern photosynthesis pathways rise from 25% completeness at 3 Ga to 50% complete at 2.5 Ga is consistent with this. The inferred late origin of anoxygenic photosynthesis is surprising but consistent with some other studies [58,59], although there is no consensus [60]. If the earliest photosynthesizers were anoxygenic [61], our analysis suggests they are not direct ancestors of modern anoxygenic photosynthetic bacteria, which are mainly found in rare reducing lakes. Hypothesized Archean lineages would have inhabited an ocean with high concentrations of electron donors (e.g. Fe^2+^). With the GOE and the creation of stratified ocean redox conditions, and ultimately an oxygenated ocean, the concentrations of those electron donors declined markedly [62]. More recently evolved lineages well adapted to much lower electron donor concentrations conceivably outcompeted hypothesized Archean lineages.

Our results indicate that methanotrophic pathways attained completeness relatively recently, congruent with the patchy phylogenetic distribution of this metabolism in modern bacteria [63]. Our inferences that other methane metabolic pathways evolved early (figure 2*b*) is consistent with previous work on the acetyl-CoA pathway [64–66]. Sedimentary geochemical evidence for methanogenesis extends to 3.46 Ga [67]. Although it is impossible to exclude abiotic methanogenesis [8], these earliest geochemical records are younger than our 3.77 Ga (Eoarchean) estimate for the origin of the mcrD subunit; mcrD may not, however, be essential for methane production [68]. The inferred age of methanogenesis is also consistent with independent phylogenetic dating of methanogenic clades [69] and geochemical inferences of extensive methane cycling from very negative organic carbon isotopes 3.47 Ga onwards [70–73] and from mass-independent fractionation of sulfur isotopes between 2.5 and 3.5 Ga [74]. Furthermore, it should be noted that there is a very limited sedimentary rock record older than 3.5 Ga.

Geological evidence contemporaneous with LUCA is lacking [7,42] and so our inference of assimilatory nitrate reduction in LUCA cannot be corroborated by sedimentary geochemical evidence. However, our inference that nitrogen fixation had evolved by 3 Ga is consistent with the geochemical evidence for nitrogen fixation between 3.2 and 2.75 Ga (late Archean) [48], other reconciliation analyses [50] and geological evidence suggesting that nitrogen fixation would have been established by 2.7 Ga [42,50]. The evolution of nitrogen fixation at this time would also be coincident with evidence for increased biosphere productivity indicated by the broadened range in mass-independently fractionated sulfur isotopes of approximately 2.7–2.4 Ga [75].

Our inference that thiosulfate oxidation began to emerge between 3.5 and 2.75 Ga (figure 2*d*) is consistent with an estimate of the earliest transfer or duplication of thiosulfate oxidation enzymes at 2.88 Ga (late Archean) [76]. Although there is convincing geological evidence for sulfate reduction by 3.5 Ga [77], it is impossible to distinguish assimilatory sulfate reduction from dissimilatory sulfate reduction. Our results suggesting assimilatory sulfate reduction evolved prior to dissimilatory sulfate reduction is in contrast to previous suggestions [8,78–80], and our estimates of the origin of dsrAB are younger than those of Matteos *et al.* [76]. However, both studies are consistent in suggesting a complete dissimilatory sulfate pathway being established by 2 Ga. These results are in agreement with a recent focused analysis [46], although the inferred bacterial origin of dsrAB is in conflict with some previous work [81] implicating an archaeal origin of the pathway. Part of the reason for this disagreement may be the uncertain evolutionary history of the key genes. In our reconciliation analysis, the oldest node with high presence probability of dsrA and B is within the Proteobacteria, but a range of nodes, including some within TACK Archaea, have appreciable origination probabilities.

### (c) The reconstructed history of metabolic assembly and hypotheses of biospheric evolution

Can an inferred sequence of historical events inform our understanding of the generative processes that underlie that history? Based on a reconstructed history of metabolic evolution, we can ask which aspects of that history we might expect to observe if the process was re-run, or if it was to occur on other Earth-like planets. Some aspects of this question seem tractable, either because they follow from simple models of how biodiversity might be structured, or because the same patterns are observed repeatedly in different clades across the tree of life. For example, we would expect any tree of life to have two long branches at its base, simply because the tree is inferred from extant modern biodiversity, and so will have fewer surviving branches going backwards in time [82]. The division of prokaryotes into Archaea and Bacteria is therefore not surprising, while the long branches associated with the origin of eukaryotes would not be expected under a simple coalescent model of tree shape. Similarly, the ‘early burst’ mode of evolutionary innovation—which appears to characterize the assembly of the KEGG [32] metabolic map (figure 1)—has been reported for many clades across the tree of life [29,83] (including the recent rapid adaptation of SARS-CoV-2 to humans [84]) and might be expected to be a general feature of diversification. We would also expect that aerobic metabolism and oxygenic photosynthesis would (co-)evolve if metabolic evolution was run again, with the extent of gene transfer for the key genes of aerobic metabolism (figure 2e; [29]), the extent to which plastids have been exchanged laterally in eukaryotes [85] and the self-reinforcing relationship between the two metabolisms (with one providing the substrate for the other) arguing in favour of this conclusion [30]. Finally, it is tempting to conclude from the apparently rapid origin of cellular life on Earth (figures 3 and 4) that the origin of cells from prebiotic geochemistry is not a rate-limiting step in biospheric evolution.

While the above considerations suggest that some general conclusions can be cautiously drawn from a reconstructed history of metabolic evolution, other questions arguably remain under-determined by the available evidence. One of the key observations that Earth system science has tried to explain is the existence of a stable biosphere that cycles the elements and organic compounds needed for life and, in so doing, enhances productivity and habitability [2]. The existence of this apparently stable system is particularly noteworthy because there is no consensus theory for how, or whether, biospheric assembly and stability might be promoted at a global level. Two interesting suggestions are the entropic Gaia and Darwinized Gaia hypotheses [18,19].

In the entropic Gaia view, the biosphere tends asymptotically towards a state of greater productivity, diversity and stability, corresponding to greater informational entropy [19]. This is hypothesized to occur through a series of punctuated equilibria where the waiting time between major reorganizations increases over time, as it becomes harder to disrupt progressively more stable equilibria. However, in the history of any given biosphere, there may be steps backwards as well as forwards. The origin of new metabolisms of energy capture and resource recycling are key contributors to increasing productivity and potential triggers of reorganization.

In Darwinized Gaia (also known as ‘it’s the song not the singer’ (ITSNTS) [18,86–88]), biogeochemical cycles are units of persistence-based selection [89] that are ‘re-produced’ should they be lost (in part or whole), invoking ‘downward causation’ in which the cycle recruits new organisms to perform functional steps within it. Some cycle variants out-persist others based on cycle-level properties and come to dominate (are ‘fittest’). Those cycle-level properties could include effects on other variables (e.g. oxygen) that affect differential success.

Based upon our reconstruction of metabolic evolution, we can ask whether any of the patterns predicted by the entropic or Darwinized Gaia views are reflected in that inferred history. A clear result of our analyses is that, while metabolic innovation has continued through time, much of modern metabolism was established relatively early in Earth’s history, in the Hadean and early Archean (figure 1*a*–c), with later periods filling out pathways that, in partial form, already appear to have existed early on. This pattern might be considered compatible with the entropic Gaia view, which implies that the rate of innovation decreases, and the length of relatively stable periods increases through time as the biosphere’s metabolic repertoire accumulates. The inference of metabolic innovation in the Archean has been described previously [36] and is reminiscent of hypotheses in which organisms colonizing empty niches can experience rapid success [83].

With respect to Darwinized Gaia, we see some evidence of pathways that have followed ITSNTS dynamics. For example, the early history of nitrogen fixation suggests that different genes arose in phylogenetically distinct lineages but were combined by HGT to perform nitrogen fixation in Proteobacteria (figure 3). However, our reconstructions also make clear that other metabolisms have been associated with particular clades through deep time, including methanogenesis [43] and oxygenic photosynthesis [90]. That is, many pathways appear to have historically been associated with the same lineages that encode them today and, while HGT is widespread, donors and recipients are predominantly close relatives (figure 1*d*; [91]). Indeed, for the set of pathways that we focus on, the degree of vertical signal in key metabolisms is surprisingly high in comparison to the speed and phylogenetic distance with which antibiotic resistance and some other functions can be spread by HGT [92,93]. This variation in the propensity for transfer might reflect the number of genes required to implement a metabolism or the difficulty of integrating the required protein machinery into a new cellular context [53]. For example, methanogenesis and oxygenic photosynthesis both require substantial cellular machinery to operate endogenously [43,90] while, by contrast, electron transport chains appear to be tolerant of evolutionary mosaicism [94]. It may be that ITSNTS provides a useful description of the evolutionary dynamics of more cosmopolitan, generalist and transferable traits, but not that subset of microbial metabolism that has remained lineage-associated through time.

A selectively neutral explanation for the dynamics of cycle assembly over time is yet to be explored, but we make some suggestions here. The key point is to note that the ‘population size’ of variants for a metabolic cycle at a given point in time is likely to be low, simply because the parts list for a variant is extensive: each variant is implemented by a (local or dispersed) community of microbes that collectively carry out a series of reactions. The simplest situation would be the case in which each variant makes use of a distinct set of functionally similar but non-homologous enzymes, although these are currently known for only a minority of pathways [95]. If the number of co-occurring cycle variants is indeed low, then chance events should play a role in determining which cycle variant persists. By analogy with genetic drift, consider a set of *N* co-occurring cycle variants that have the same propensity to persist. Then, the probability that a given variant is the one that persists is 1 */N*. For cycles with at most a few co-occurring variants, it will be difficult to reject the possibility that the persister was fixed by chance alone (e.g. with *P*_fixation_ = $\frac{1}{2}$ or $\frac{1}{3}$ for a cycle variant with two or three co-occurring variants, respectively).

The inference that many metabolic pathways have not risen monotonically through time, but instead meander in frequency prior to completion (figure 2) might be seen as evidence for the role of chance in global metabolic evolution. In this neutral model, the stability of the biosphere emerges because of a process akin to constructive neutral evolution [96,97]. As cycles are locked in (variants winnowed to a single ultimate persister, which has occurred at different times for different metabolisms, figure 2), they alter the playing field upon which further rounds of ‘biospheric drift’ or persistence-based selection can occur. Evidently, a simple neutral explanation for pathway or cycle dynamics would be most appropriate in cases where co-occurring cycle variants have similar intrinsic propensities for persistence, while persistence-based selection would be most powerful in cases where cycle variants were in direct competition [30]. If selection at the biosphere level is indeed generally weak, we would expect the observed history to be driven by evolution at lower levels—for example, the immediate adaptive value of new genes or metabolic pathways to the organisms that encode them.

## 5. Conclusion

Overall, our reconstruction of metabolic evolution emphasises the heterogeneity of the process, through time, across lineages and among different metabolic pathways. The rate of metabolic evolution appears to have been higher earlier in Earth’s history [15,36], and the rate of pathway assembly has varied between pathways cycling different key elements. An anaerobic carbon cycle was established early in life’s evolution, whereas the biological cycling of elements such as nitrogen and sulfur began later, though nonetheless early in the Earth’s history. HGT has been a major force in microbial evolution, with Bacteria having higher rates than Archaea during the history of life. Our analyses also suggest that some pathways have been affected unevenly by HGT. The question of whether there is a simple high-level explanation for the inferred history is a complicated one, and some aspects of life’s evolution appear to be compatible with each of the models discussed. Definitively testing between them may require observation of multiple biospheres—for example, on exoplanets [98]—or simulations that explore their differing predictions, and we suggest that there is an opportunity to consider the role of chance and determinism in evolutionary accounts of biospheric evolution.

## Data Availability

The data associated with this article are available on figshare [38]. A high resolution version of figure 3 is available at [44].
